# The association between osteoporosis medications and lowered all-cause mortality after hip or vertebral fracture in older and oldest-old adults: a nationwide population-based study

**DOI:** 10.18632/aging.203927

**Published:** 2022-03-01

**Authors:** Chia-Chun Li, Jason C. Hsu, Fu-Wen Liang, Yin-Fan Chang, Ching-Ju Chiu, Chih-Hsing Wu

**Affiliations:** 1 Institute of Allied Health Sciences, College of Medicine, National Cheng Kung University, Tainan, Taiwan; 2 Department of Family Medicine, College of Medicine, National Cheng Kung University, Tainan, Taiwan; 3 International PhD Program in Biotech and Healthcare Management, College of Management, Taipei Medical University, Taipei, Taiwan; 4 Clinical Data Center, Office of Data Science, Taipei Medical University, Taipei, Taiwan; 5 Research Center of Data Science on Healthcare Industry, College of Management, Taipei Medical University, Taipei, Taiwan; 6 Department of Public Health, College of Health Sciences, Kaohsiung Medical University, Kaohsiung, Taiwan; 7 Department of Family Medicine, National Cheng Kung University Hospital, College of Medicine, National Cheng Kung University, Tainan, Taiwan; 8 Institute of Gerontology, College of Medicine, National Cheng Kung University, Tainan, Taiwan; 9 Clinical Big Data Research Center, Taipei Medical University Hospital, Taipei Medical University, Taipei, Taiwan

**Keywords:** osteoporosis, hip fracture, vertebral fracture, ageing, oldest-old adult

## Abstract

Background: Osteoporotic fracture is a common public-health problem in ageing societies. Although post-fracture usage of osteoporosis medications may reduce mortality, recent results have been inconsistent. We aimed to examine associations between osteoporosis medication and mortality in older adults, particularly oldest-old adults (>=85 years old).

Methods: Participants aged 65 years old and older newly diagnosed with both osteoporosis and hip or vertebral fractures within 2009-2017 were recruited from the records of 23,455,164 people in Taiwan National Health Insurance Research Database (NHIRD). Osteoporosis medication exposure was calculated after the first-time ambulatory visit with newly diagnosed osteoporosis. Mortality and its specific causes were ascertained from Cause of Death Data. Patients were followed until death or censored at the end of 2018.

Results: A total of 87,935 participants aged 65 years old and over (73.4% female), with a mean 4.13 follow-up years, were included. Taking medication was associated with significantly lower risk of mortality (hip fracture HR 0.75, vertebral fracture HR 0.74), even in the oldest-old adults (hip fracture HR 0.76, vertebral fracture HR 0.72), where a longer duration of taking osteoporosis medication was associated with lower all-cause mortality. Specific causes of mortality were also significantly lower for participants taking osteoporosis medication (cancer HR 0.84 in hip fracture, 0.75 in vertebral fracture; cardiovascular disease HR 0.85 in hip fracture, 0.91 in vertebral fracture).

Conclusions: Osteoporosis medication after hip or vertebral fracture may reduce mortality risk in older adults, notably in oldest-old adults. Encouraging the use of post-fracture osteoporosis medication in healthcare policies is warranted.

## INTRODUCTION

Osteoporosis is an alarming disease in older adults that may cause health hazards such as increased economic burdens, morbidity, and decreased health-related quality of life [1–4]. The prevalence of osteoporosis is rapidly increasing among the older population [5]. In a WHO report, the burden of osteoporotic fractures in 2002 was 2.8 million disability-adjusted life years (DALYs), which is more than that for hypertension and slightly less than that for diabetes mellitus or chronic obstructive pulmonary diseases [2]. Many patients who have had a diagnosed fracture have never been diagnosed with osteoporosis, therefore, closing the gap of osteoporosis treatment is important [6]. This situation is primarily due to the fact that osteoporosis symptoms are often not recognized until a fracture occurs [7]. Osteoporotic fractures, especially those of the hip and vertebra [8–10], are associated with an increased risk of death [11]. Early detection of high risk for osteoporotic fractures is important; however, post-fracture management, especially interventions intended to lower mortality, is an emerging public health issue in rapidly ageing societies.

Both observational studies [12–15] and a randomized trial study [16] found that osteoporosis medication can significantly reduce mortality. A systematic review supported this finding [17] and showed a tendency for such medications to reduce the risk of cardiovascular mortality [18]. However, in a recent meta-analysis study of randomized trials [19], although taking osteoporosis medication reduced the risk of fracture, it did not lower overall mortality rates and without discussing the oldest-old patients separately. These inconsistent findings may derive from unmet situations, such as short follow-up times, no adjustments for differences at baseline, no comparisons of different fracture sites, a limited number of older participants, and very few studies focusing on the oldest-old adults [20]. As such, the study participants may not have represented the entire population, which is also a limitation of any observational study or randomized study design. Therefore, a mega-databank or real-world population may be preferred to overcome these study design limitations. In addition, although the effects of osteoporosis medication on the risk of specific causes (such as cancer, cardiovascular disease, etc.) of death has become an emerging issue, to the best of our knowledge, it has never been reported.

The primary objective of this real-world evidence mega-study was to determine, via a nationwide databank, the association between osteoporosis medication and mortality in older and oldest-old adults who had suffered from a hip or vertebral fracture and had long-term follow-up. The secondary objective was to focus on the novel investigation of specific causes of death (e.g., cancer and cardiovascular disease) in older and the oldest old adults.

## RESULTS

**Figure 1:**
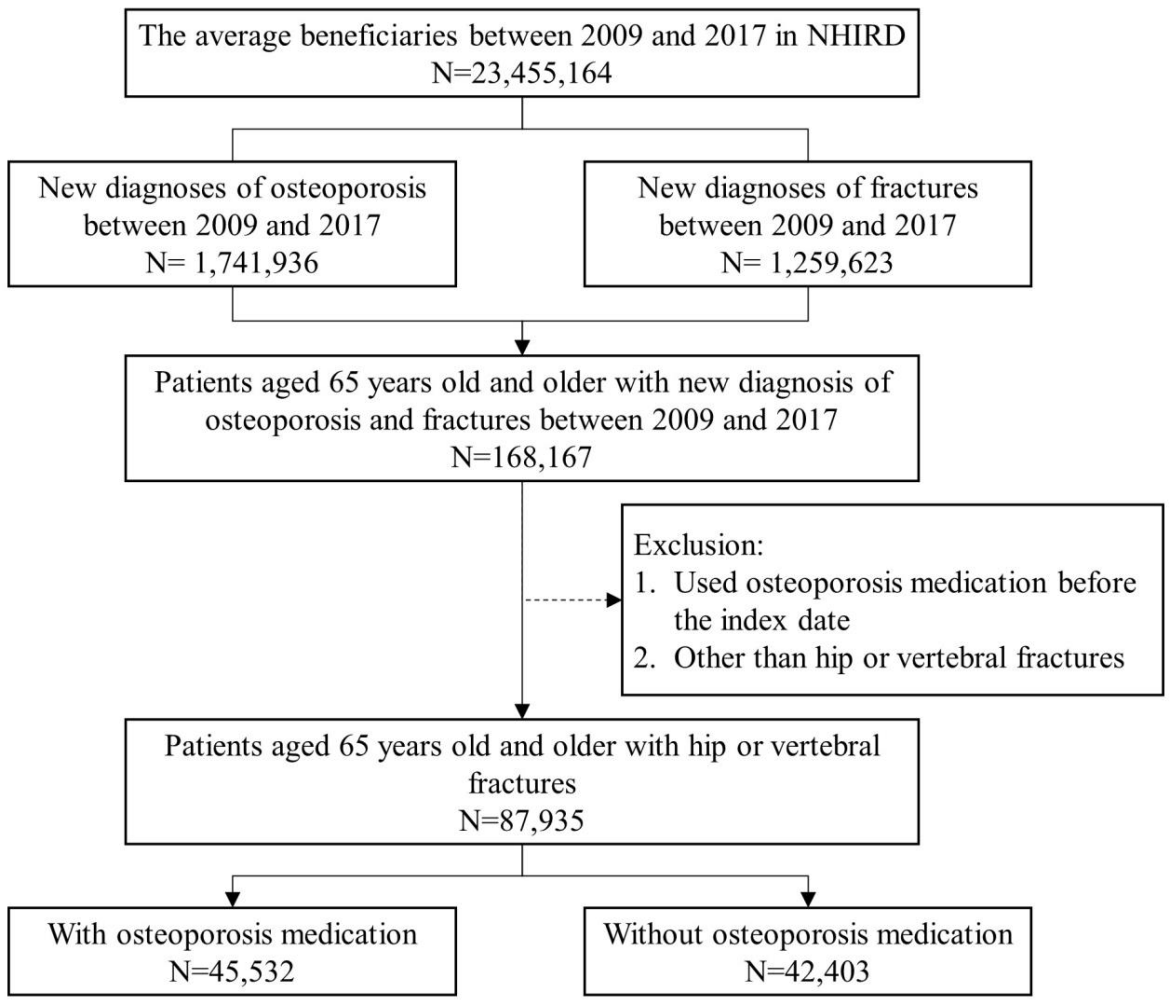
Characteristics of eligible subjects from the National Health Insurance Research Databank (NHIRD) cohort.

Figure 1 shows the participants considered eligible for the study. The number of beneficiaries from 2009 to 2017 was 23,455,164 annually, on average [21]. There were 168,167 patients aged 65 years and older who were newly diagnosed with both osteoporosis and a fracture between 2009 and 2017. A total of 87,935 participants aged 65 years and older with hip or vertebral fractures were analyzed in this study. As shown in Table 1, the participants were predominantly female (73.4%), with 51.8% taking osteoporosis medication. The mean age of the participants was 78.2 years old (SD 5.98); the mean CCI score was 2.25 (SD 2.09); the mean follow-up years was 4.13 years (SD 2.51), and the mean duration of taking osteoporosis medication was 1.26 years (SD 1.32). Of these, 42.7% of the participants died during the follow-up period. The basic characteristics of the groups with or without osteoporosis medication were significantly different.

**Table 1:** Baseline characteristics of osteoporotic fracture participants aged 65 years old and older.

	**Participants, No. (%)**			***p* value**
	**Total** **(n=87,935)**	**With osteoporosis medication** **(n=45,532)**	**Without osteoporosis medication** **(n=42,403)**	
Sex				<0.001
Male	23418 (26.6)	9162 (20.1)	14256(33.6)	
Female	64517 (73.4)	36370 (79.9)	28147(66.4)	
Age, mean (SD), y	78.20 (5.98)	77.94 (5.91)	78.48 (6.03)	<0.001
Charlson Comorbidity Index score, mean (SD)	2.25 (2.09)	2.13 (2.02)	2.39 (2.16)	<0.001
Follow-up year, mean (SD), y	4.13 (2.51)	4.64 (2.50)	3.57 (2.41)	<0.001
Duration of osteoporosis medication, mean (SD), y	1.26 (1.32)	1.26 (1.32)	-	-
Survival status				<0.001
Survival	50394 (57.3)	27429 (60.2)	22965 (54.2)	
Death	37541 (42.7)	18103 (39.8)	19438 (45.8)	

Data are n (%) or mean±SD. *p* value for t-test on continuous variables, or χ^2^ test on categorical variables.

In Table 2, after adjusting for sex, age, and the CCI score, the participants aged 65 years and older taking medication were associated with a significantly lower risk of either hip fracture (HR 0.75, 95% CI 0.73-0.77) or vertebral fracture (HR 0.74, 95% CI 0.72-0.76). For the subgroup of the oldest-old adults (aged 85 and older), the results still showed consistently lower mortality risk in the hip fracture (HR 0.76, 95 % CI 0.72-0.79) and vertebral fracture groups (HR 0.72, 95 % CI 0.68-0.76). The Kaplan-Meier survival curve plots for adults aged >=65 years old and >=85 years old are shown in the Online-Only Supplements (Supplementary Figures 1, 2).

**Table 2:** Multivariate Cox proportional hazard analyses of the association between hip or vertebral fracture and mortality in older adults (≥65 years old) and oldest-old adults (≥85 years old).

	**HR (95% CI)**			
	**Hip fracture (95% CI)**		**Vertebral fracture (95% CI)**	
	**Older adults** **(n=45,367)**	**Oldest-old adults** **(n=13,120) ^a^**	**Older adults** **(n=43,118)**	**Oldest-old adults** **(n=7,670) ^a^**
Gender (ref. Male)	1.00	1.00	1.00	1.00
Female	0.70(0.68-0.72) ***	0.77(0.74-0.81) ***	0.66(0.64-0.69) ***	0.80(0.75-0.85) ***
Age, y	1.08(1.08-1.09) ***	-	1.09(1.08-1.09) ***	-
Charlson Comorbidity Index score	1.12(1.11-1.12) ***	1.07(1.06-1.08) ***	1.13(1.12-1.14) ***	1.06(1.05-1.08) ***
Osteoporosis medication				
Without medication	1.00	1.00	1.00	1.00
With medication	0.75(0.73-0.77) ***	0.76(0.72-0.79) ***	0.74(0.72-0.76) ***	0.72(0.68-0.76) ***

Abbreviations: HR, hazard ratio; CI, confidence interval.

***p < 0.001.

^a^According to the coding book of the National Health Insurance Research Databank (NHIRD), all adults aged 85 years and older are defined as ‘**≥**85’, with no adjustment for age in the oldest-old group.

In Table 3, it was shown that a longer duration of osteoporosis medication was associated with lower mortality in patients experiencing either a hip or vertebral fracture. When the medication duration was over two years, the reduction in mortality was nearly half that of those who were not taking medication (HR=0.51 in the hip fracture group and HR= 0.56 in the vertebral fracture group).

**Table 3:** Multivariate Cox proportional hazard analyses of the association between hip or vertebral fracture and mortality in older adults (≥65 years old) with different osteoporosis medication duration.

	**HR (95% CI)**	
	**Hip fracture** **(n=45,367)**	**Vertebral fracture** **(n=43,118)**
Gender (ref. Male)	1.00	1.00
Female	0.71(0.69-0.74) ***	0.68(0.66-0.70) ***
Age, y	1.08(1.08-1.09) ***	1.09(1.08-1.09) ***
Charlson Comorbidity Index score	1.12(1.11-1.12) ***	1.13(1.12-1.14) ***
Osteoporosis medication duration, y		
Without medication	1.00	1.00
With medication < 1 year	0.90(0.87-0.92) ***	0.93(0.89-0.96) ***
1 <= With medication < 2 years	0.67(0.64-0.71) ***	0.66(0.63-0.70) ***
2 <= With medication < 3 years	0.51(0.48-0.56) ***	0.56(0.52-0.61) ***
3 years <= With medication	0.35(0.32-0.38) ***	0.34(0.31-0.37) ***

Abbreviations: HR, hazard ratio; CI, confidence interval.

***p < 0.001.

After re-considering the immortal time bias inference [22], the participants taking medication still exhibited lower mortality risk in both the hip fracture (HR 0.89, 95% CI 0.87-0.92) and vertebral fracture groups (HR 0.87, 95% CI 0.84-0.90) (Supplementary Table 1). Furthermore, we adjusted the baseline covariates based on the IPTW with the PS, the results of which were consistent with the unadjusted Cox proportional models (HR 0.75 in the hip fracture group and 0.74 in the vertebral fracture group) (Supplementary Table 2).

Cancer and cardiovascular disease are the two major causes of death in older adults. The competing risk of a specific cause of death is presented in Table 4. As shown in the table, participants taking osteoporosis medication showed significantly lower risks of a specific cause of death from both cancer (HR 0.84 in the hip fracture group and 0.75 in the vertebral fracture group) and cardiovascular disease (HR 0.85 in the hip fracture group and 0.91 in the vertebral fracture group).

**Table 4:** Competing risk survival analysis of associations between hip or vertebral fracture and two major cause-specific mortalities in older adults (≥65 years old).

	**HR ^a^**			
	**Hip Fracture** **(n=45,367)**		**Vertebral Fracture** **(n=43,118)**	
	**Cancer**	**Cardiovascular disease**	**Cancer**	**Cardiovascular disease**
Female (ref. Male)	0.66***	1.07	0.63***	0.89**
Age	1.01***	1.08***	1.01***	1.11***
Charlson Comorbidity Index score	1.18***	1.02**	1.19***	1.04***
Osteoporosis medication				
Without medication	1.00	1.00	1.00	1.00
With medication	0.84***	0.85***	0.75***	0.91*

Abbreviations: HR, hazard ratio; CI, confidence interval.

*p < 0.05, **p < 0.01, ***p < 0.001.

^a^Hazard ratio (HR) was analyzed using a multivariate Cox proportional hazard analyses with a cumulative incidence function.

## DISCUSSION

This real-world, nationwide, mega-database study provides substantial evidence of an association between osteoporosis medication usage after hip or vertebral fractures and all-cause mortality in older and oldest-old adults. The main result showed that osteoporosis medication is significantly associated with decreased risk for all-cause mortality after hip or vertebral fractures, which agrees with the findings of previous studies [12–15, 23], even for the oldest-old adults (>= 85 years old). As with all-cause mortality, the specific causes of cancer or cardiovascular disease were also lowered comparably.

Population ageing is an emerging trend worldwide that presents challenges for healthcare systems and economies. Taiwan will become a super-aged society by 2025 and ranks near the top globally in terms of a rapidly ageing demographic. Therefore, not only do these first-hand findings from Taiwan support the necessity of post-fracture osteoporosis medication, but also help inform public health strategies in ageing societies.

The burden of osteoporotic fracture is higher than that for hypertension [2]; however, osteoporosis is always given less attention than other chronic diseases by health professionals and the general population. In this study, we found that although the health insurance system in Taiwan provides equal coverage to everyone, nearly half of the older adults did not receive osteoporosis medication despite having been diagnosed with both osteoporosis and a fracture. This result is similar to the previous study [24]. Another retrospective study showed a similar result, only 33% of patients received osteoporosis medication after their fracture [25]. Although the contraindications or availability of medication may partially explain the treatment gap, to improve awareness of medication usage after osteoporotic fractures is an unmet challenge [6]. In addition, osteoporosis medication showed a tendency to reduce cardiovascular mortality [18], and sometimes was used as a cancer bone metastasis regimen [26]; thus, these interventions may provide benefits beyond osteoporosis treatment, especially in older, frailer adults [17]. In summary, this mortality study highlights the importance of older adults getting the right treatment for osteoporosis at the right time.

Studies by Center et al. [14] and Yu et al. [15] also concluded that osteoporosis medication can significantly reduce the risk of mortality in older adults. Center et al. and Yu et al. targeted osteoporosis medications, bisphosphonates and hormone therapy in Center’s study, and bisphosphonates, raloxifene, calcitonin and teriparatide in Yu’s study. In Lyles et al.'s study [16], zoledronic acid has significant impact on all-cause mortality. In contrast, the types of osteoporosis medication in those studies [14–16] were not comprehensive as our study concomitantly. We also analyzed all Nation participants who satisfied the inclusion criteria from the NHIRD and focused on both hip and vertebral fractures with follow-ups on the death status and specific cause of death over an extended period from 2009-2018. In controlling the differences at baseline, we further adjusted the Cox model using the IPTW by PS for the baseline covariates to estimate the causal treatment effects [27]. By so doing, the findings of this study satisfy several gaps in the literature review, e.g., the representativeness of nation population, two types of fracture, compatibly longer follow-up times, and considering the impact of co-morbidities at the baseline as possible, and nearly all types of osteoporotic medications.

In 2018, the coverage rate among older adults in the NHI system was 99.7% [21]; accordingly, the eligible participants in this study represented the entire ageing population in Taiwan. The NHIRD in Taiwan has been used to study public health issues among the whole population over long periods of time, such as the prevalence or incidence of specific diseases and entire medical records, with high reliability and representativeness. Accordingly, we were able to easily observe the nationwide prevalence of osteoporotic fractures, as well as the details of all osteoporosis medication records. As such, this study derived from a nationwide databank provides comprehensive real-world evidence with high reliability.

This study had several strengths that should be emphasized. Firstly, in order to observe effects on the oldest-old adults, which differed from previous research, we analyzed participants aged 85 years and older. To our knowledge, this is the first study to discuss osteoporosis-medication effects among oldest-old adults, who are often underrepresented in randomized controlled trials or observational studies [28]. Most importantly, this is the first population-level observational study to discuss the effects of osteoporosis medications with a large representative sample. Secondly, the immortal time bias was considered to reconfirm the effects of treatment with osteoporosis medications. Thirdly, the database was derived from the NHIRD, which is population-representative and provides reliable real-world evidence. This study could overcome limitations of previous observational or cohort studies and provide reliable comprehensive real-world evidence. Fourthly, older or oldest-old adults have typically been excluded from most randomized clinical trials related to osteoporosis. Therefore, there is little evidence of the efficacy of treatment duration. The substantial findings from this study address this issue. This study aims to raise the importance of awareness about treatment of osteoporosis. Through aggressive and timely treatment, lowered mortality could be observed. Finally, there are some emerging issues related to specific causes of death after treatment with osteoporosis medications. For example, calcitonin increases the risk of cancer [29], or strontium ranelate which leads to a higher risk of heart attack than placebos [30]. Be that as it may, these regimens are not reimbursed for clinical use by the TFDA. On the other hand, the competing risk associated with cancer and cardiovascular disease can mitigate these emerging issues associated with osteoporosis medications. We not only focused on all-cause mortality, but also discussed cancer and cardiovascular mortality with cause-specific hazards in a Cox proportional hazard model different from previous studies.

Nevertheless, this study still has some limitations. The NHIRD has unmeasured potential confounders, e.g., lifestyle or socioeconomic status, which might impact disease status. To mitigate this concern, we calculated the CCI score within 1 year before the first hospitalization with a hip or vertebral fracture to adjust for the impact of the existing disease status. Furthermore, the confounding influence of relatively low prevalence (< 5%) of alcohol and smoking usage among older adults in Taiwan [31] may be minimized. The sensitivity analysis for unmeasured confounding, the E-value [32], was 1.73-1.76 which can reflect the stability of the results in this study. The T-score for bone mineral density (BMD) was not available for adjustment. Based on the NHI regulations, a measure of BMD is necessary for the diagnosis of osteoporosis as well as for reimbursement for osteoporosis medications if the T-score is less than -2.5, the same with another study suggestion [33]. Therefore, participants diagnosed of fractures are in consistency with similar status of BMD. Lastly, this study did not exclude changes in osteoporosis medication during the follow-up period. Whether the unique mechanisms of different types of osteoporosis medications can exert a different impact on mortality is interesting and requires further study in the future.

In conclusion, the findings of this study suggest that in older and oldest-old adults with osteoporotic hip or vertebral fractures, the usage of osteoporosis medication may reduce all-cause mortality risk, especially with respect to patients with cancer and cardiovascular disease. Therefore, it is plausible to encourage the use of post-fracture osteoporosis medication in the health policies of ageing societies.

## MATERIALS AND METHODS

### Data source

The Taiwanese National Health Insurance (NHI) program was launched in 1995, and in 2018, it covered 99.8% of Taiwan’s 23 million people [21]. The National Health Insurance research database (NHIRD) was established to archive and store all NHI medical claims nationwide. It contains information concerning disease diagnoses as well as detailed information on healthcare for ambulatory patients and inpatients. Disease diagnosis is assigned according to the International Classification of Diseases, 9th and 10th Revisions, and the Clinical Modification (ICD-9-CM and ICD-10-CM) diagnostic codes. The datasets provided by the Health and Welfare Data Science Center (HWDC), Ministry of Health and Welfare (MOHW), were also included along with the Ambulatory Care Expenditures by Visits and Inpatient Expenditures by Admissions. In this study, we used the NHIRD to analyze the association between osteoporosis-related fractures and osteoporosis medication in Taiwan. Details of the Ambulatory Care Orders were collected to record the duration of osteoporosis medication, while the Cause of Death Data were obtained from the National Death Registry to identify the death status and specific cause of death.

### Study participants

The osteoporosis cohort included patients aged 65 years and older between 2009 and 2017 who were newly diagnosed with osteoporosis (ICD-9-CM: 733.0 and 733.1, ICD-10-CM: M80 and M81) at least twice in outpatient records. The fracture cohort comprised patients who were newly diagnosed with either a hip fracture (ICD-9-CM: 820 and 733.14, ICD-10-CM: S72) or a vertebral fracture (ICD-9-CM: 805.2-805.9 and 733.13, ICD-10-CM: S22.0, S22.1, and S32.0-S32.2) once in an inpatient record, the definition was based on the previous study on hip fracture using Taiwan’s NHI claims [31, 34]. Participants who satisfied both cohorts were enrolled, and their death status was followed up until December 31, 2018. To identify those who were newly diagnosed starting in 2009, we applied a backward washout to 2008. The index date was the discharge date of the first hospitalization with a hip or vertebral fracture. The main study outcome was all-cause mortality. For all participants who died of any underlying causes of death, information on months of survival from the index date was provided from the Cause of Death Data [35], and the specific cause of death defined by the ICD-10-CM code was the 2^nd^ outcome.

### Osteoporosis medication exposure

In this study, medication exposure was defined as the usage of osteoporosis medications approved by the Taiwan Food and Drug Administration (TFDA), including alendronate, risedronate, ibandronate, zoledronic acid, denosumab, raloxifene, bazedoxifene, calcitonin, and teriparatide, but excluded patients using the osteoporosis medication for cancer-related treatments (such as high dosing frequency of zoledronic acid or denosumab). Patients who used osteoporosis medication before the index date were excluded to minimize any residual effects of the medications. To evaluate the influence of medication adherence on all-cause mortality, the duration of taking osteoporosis medication was calculated from the total drug exposure time after initiating osteoporosis medication in the ambulatory visits until the end of 2017. To minimize immortal time bias, the day of initiating medication, rather than the day of diagnosis, was used to calculate the follow-up interval [22]. Based on the methodology, the survival effect will be estimated in a more conservative manner.

### Covariates

Demographic characteristics, including gender and age, but no lifestyle information, were collated when the patient was newly diagnosed with osteoporosis. However, mortality after an osteoporotic fracture may be attributable to a patient’s known health factors. These health factors might be related to the major illness with mortality but unmeasured in NHIRD. Therefore, we calculated the Charlson Comorbidity Index (CCI) score to adjust the impact of major illness before fracture. The CCI score [36] was calculated using the ambulatory visit diagnoses of CCI diseases with the ICD-9/10-CM codes within 1 year before the index date.

### Statistical analysis

This study followed the Strengthening the Reporting of Observational Studies in Epidemiology (STROBE) reporting guidelines for observational studies.

Statistical analyses were performed using *t*-tests to compare the continuous variables, and χ^2^ tests were used for the categorical variables. The multivariable Cox proportional hazard model was used to estimate the hazard ratio (HR) with a 95% confidence interval (CI) in those with and without osteoporosis medication based on several categorical variables. A subgroup analysis based on age 85 and older was similarly performed. To address the inference by indication at baseline, we adjusted the Cox model using inverse probability of treatment weighting (IPTW) with a propensity score (PS) for the baseline covariates [37], such as age, sex, CCI score. These baseline covariates were generally known risk factors for osteoporotic fracture and the measurable confounders in NHIRD. Competing risk of a specific causes of death was analyzed using the cause-specific hazard in the Cox proportional hazard model. A two-sided probability value of 0.05 was used to indicate statistical significance. All data were analyzed with SAS^®^ software, version 9.4 (SAS Institute Inc., Cary, NC, USA).

### Data availability

The data used in this study were requested from the Health and Welfare Data Science Center. The request is subject to approval by the Department of Statistics, Ministry of Health and Welfare, Taiwan. For ethical and privacy reasons, the data only were accessed and statistically analyzed at the Health and Welfare Data Science Center.

## Supplementary Material

Supplementary Figures

Supplementary Tables
