# Supplementary material for: The association between osteoporosis medications and lowered all-cause mortality after hip or vertebral fracture in older and oldest-old adults: a nationwide population-based study
Source: Aging (Albany NY). 2022 Mar 1;14(5):2239–51. doi: 10.18632/aging.203927 (PMC8954959; doi:10.18632/aging.203927)
Supplement: Supplementary Figures [file aging-14-203927-s001.pdf]

SUPPLEMENTARY FIGURES

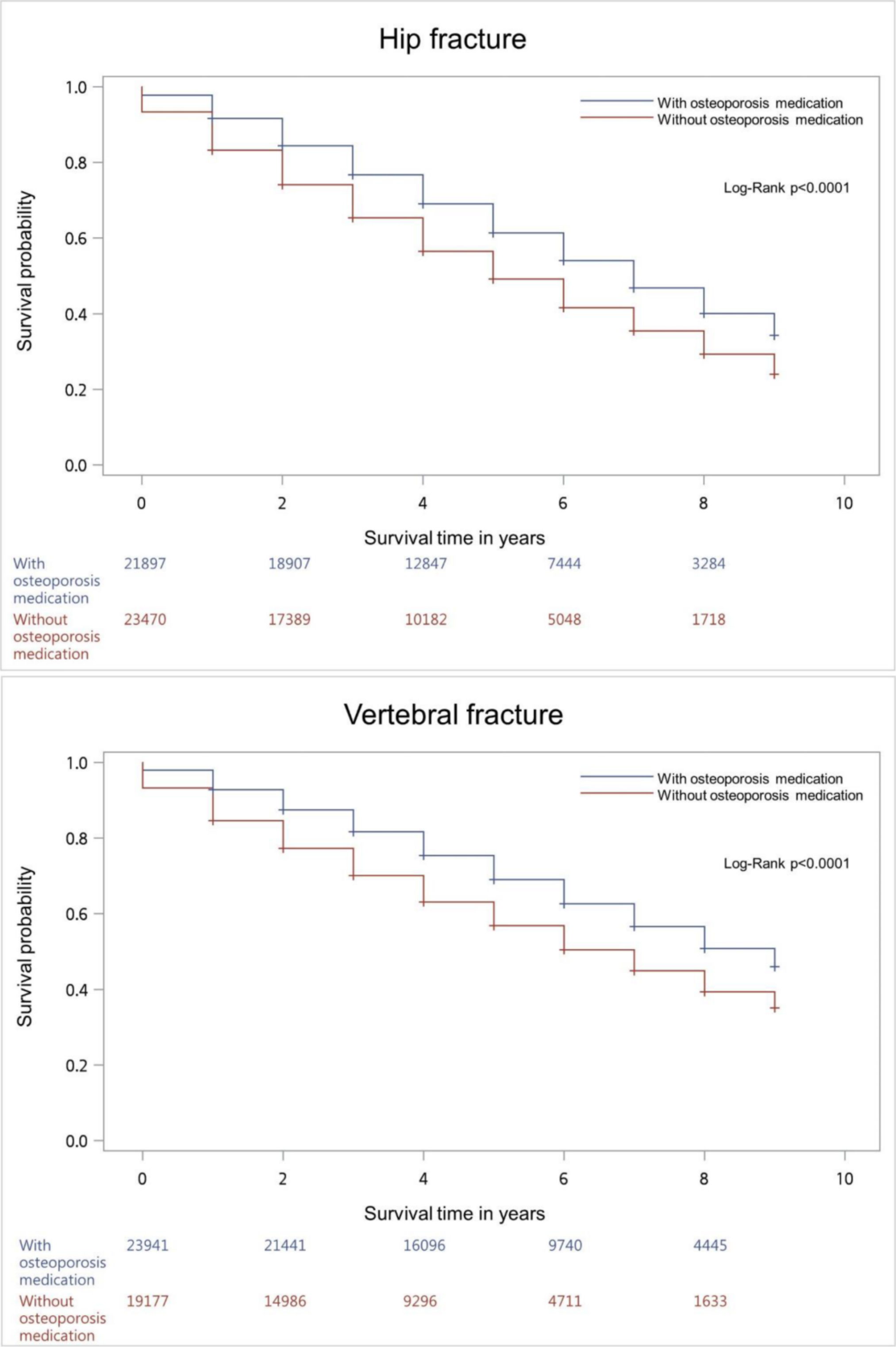

Supplementary Figure 1. Kaplan-Meier survival curve for adults aged 65 years old and older.

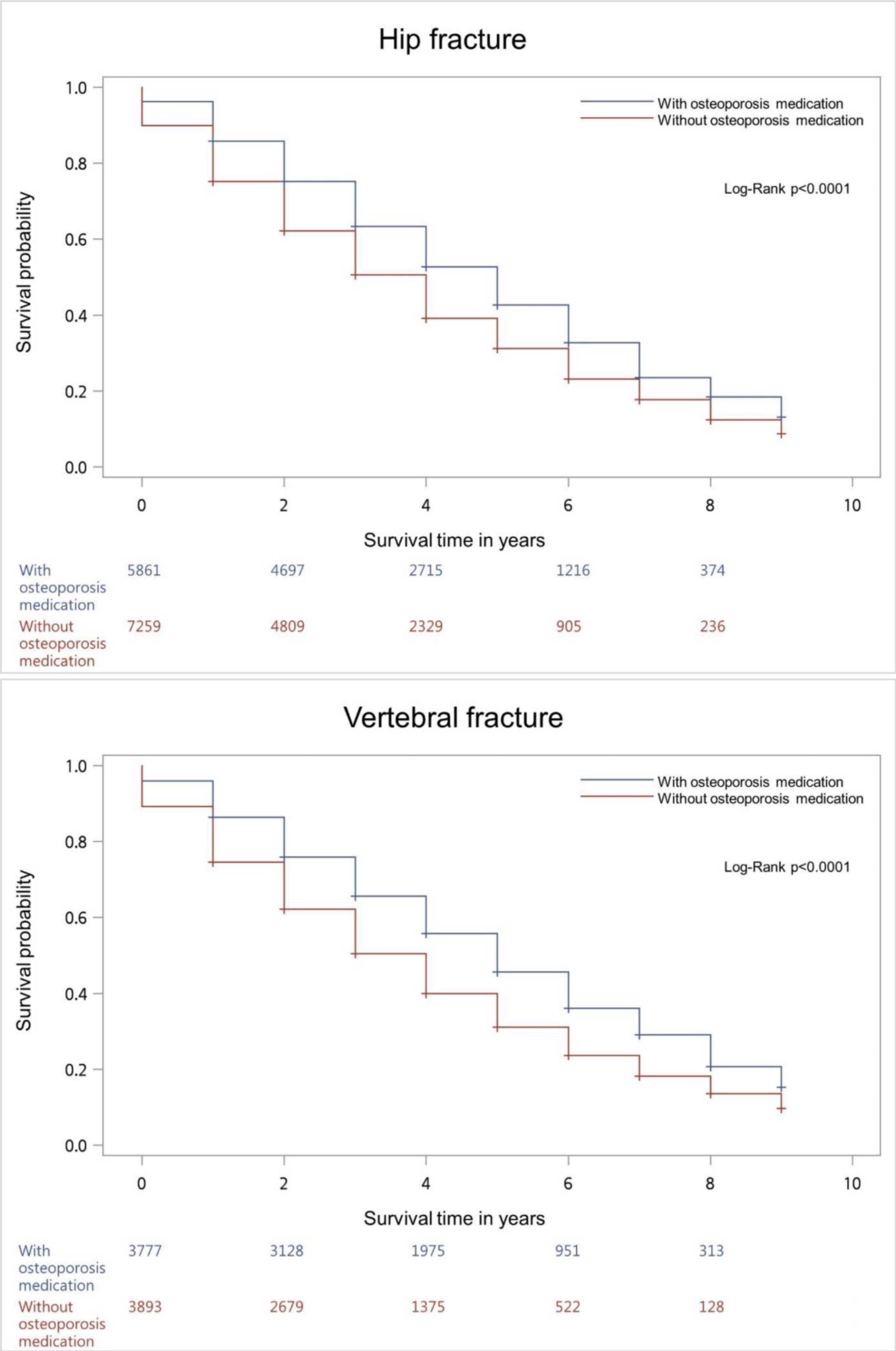

Supplementary Figure 2. Kaplan-Meier survival curve for adults aged 85 years old and older.
