# Supplementary material for: The association between osteoporosis medications and lowered all-cause mortality after hip or vertebral fracture in older and oldest-old adults: a nationwide population-based study
Source: Aging (Albany NY). 2022 Mar 1;14(5):2239–51. doi: 10.18632/aging.203927 (PMC8954959; doi:10.18632/aging.203927)
Supplement: Supplementary Tables [file aging-14-203927-s002.pdf]

SUPPLEMENTARY TABLES

Supplementary Table 1. Associations between hip or vertebral fracture and mortality in adults 65 years of age and older by multivariate Cox proportional regression adjusted for immortal time bias.

	Hip fracture N=45,367	Vertebral fracture N=43,118
	**HR (95% CI)**	**HR (95% CI)**
Gender (ref. Male)	1.00	1.00
Female	0.70(0.68-0.72) ***	0.66(0.64-0.68) ***
Age	1.08(1.08-1.09) ***	1.09(1.08-1.09) ***
Charlson Comorbidity Index (CCI) score	1.12(1.11-1.12) ***	1.13(1.12-1.14) ***
Osteoporosis medication		
Without medication	1.00	1.00
With medication	0.89(0.87-0.92) ***	0.87(0.84-0.90) ***

Abbreviations: HR, hazard ratio; CI, confidence interval. ****p*<0.001.

Supplementary Table 2. Cox proportional hazard analyses adjusted by IPTW with PS of the association between hip or vertebral fracture and mortality of adults aged 65 years old and older.

	Hip fracture N=45,367	Vertebral fracture N=43,118
	**Adjusted HR (95% CI) [a]**	**Adjusted HR (95% CI) [a]**
Gender (ref. Male)	1.00	1.00
Female	0.70(0.68-0.72) ***	0.66(0.64-0.68) ***
Age	1.08(1.08-1.09) ***	1.09(1.08-1.09) ***
Charlson Comorbidity Index (CCI) score	1.12(1.11-1.12) ***	1.13(1.12-1.14) ***
Osteoporosis medication		
Without medication	1.00	1.00
With medication	0.75(0.73-0.77) ***	0.74(0.72-0.76) ***

Abbreviations: HR, hazard ratio; CI, confidence interval. ****p*<0.001.

[a]Adjusted hazard ratio (95% confidence interval) was calculated by using multivariable Cox proportional regression and adjusted by inverse probability of treatment weighting (IPTW) with propensity score (PS). Propensity scores were calculated by generating a logistic regression model that included all baseline characteristics to predict the probability of each patient survival status.
